# Molecular Target Discovery and Systemic Mechanism Analysis of Teriflunomide for Dry Eye Disease

**DOI:** 10.3390/cimb48050492

**Published:** 2026-05-09

**Authors:** Yang Chen, Weiran Lin, Wei Feng, Wenyuan Li, Lianhao Song

**Affiliations:** 1 State Key Laboratory of Ophthalmology, Zhongshan Ophthalmic Center, Sun Yat-Sen University, Guangdong Provincial Key Laboratory of Ophthalmology and Visual Science, Guangzhou 510060, China; cheny2398@mail2.sysu.edu.cn (Y.C.);; 2 State Key Laboratory of Medical Neurobiology, Institute for Translational Brain Research, MOE Frontiers Center for Brain Science, Fudan University, Shanghai 200032, China; 3 Zhongshan School of Medicine, Sun Yat-Sen University, Guangzhou 510080, China

**Keywords:** dry eye disease, teriflunomide, virtual cell simulation, multi-target mechanisms, computational pharmacology, molecular dynamics simulation

## Abstract

Background: Dry eye disease (DED) is a multifactorial ocular surface disorder characterized by tear film instability, inflammation, and neurosensory abnormalities. Current therapies remain limited by slow onset and suboptimal efficacy. Teriflunomide, an immunomodulatory agent approved for multiple sclerosis, has shown therapeutic potential in DED, but its multi-target mechanisms remain unclear. Methods: We employed an integrated computational and transcriptomic framework combining ADMET profiling, multi-dataset transcriptomic integration, and single-cell RNA sequencing (scRNA-seq) to identify disease-relevant targets. Candidate genes were further refined through molecular docking and 50 ns molecular dynamics (MD) simulations. The AetherCell virtual cell model was applied to evaluate both the concordance between target perturbation and drug-induced responses and the potential mechanistic roles of candidate targets. Results: Transcriptomic integration identified 16 consensus genes across heterogeneous DED models, which were further localized to disease-relevant epithelial and immune cell populations by scRNA-seq. Molecular simulations prioritized three core targets—CTSS, STAT1, and PTGS1—based on binding stability and affinity. AetherCell simulations demonstrated that perturbation of these targets not only recapitulated teriflunomide-induced transcriptional and pathway changes but also revealed their distinct mechanistic contributions, including epithelial barrier regulation (CTSS), microvascular and lipid homeostasis (PTGS1), and inflammation suppression coupled with tissue repair (STAT1). Conclusions: Teriflunomide exerts therapeutic effects in DED through coordinated multi-target regulation involving inflammation control, barrier restoration, and tissue repair. This study provides a rationale for novel therapeutic targets in dry eye disease, establishes a paradigm for applying virtual cell modeling to elucidate drug mechanisms, and offers a bioinformatics framework for validating drug repositioning outcomes.

## 1. Introduction

Dry eye disease (DED), also known as keratoconjunctivitis sicca, has emerged as one of the most prevalent and challenging chronic ocular surface disorders in modern ophthalmology, significantly impacting the quality of life and visual function of hundreds of millions of patients worldwide [1]. As lifestyles have shifted toward increased use of digital devices and as environmental pollution and global population aging have intensified, the incidence of DED has shown a significant upward trend [2]. Recent meta-analyses indicate that the global pooled prevalence of DED is approximately 34.6%, with pronounced regional and demographic disparities [1]. In regions like Africa, prevalence can soar to 43.9%, whereas in North America, it is estimated at 20.9% [1]. Notably, age and sex are critical risk factors; women are more frequently affected than men, and the prevalence increases steadily with age, particularly in populations over 40 [1]. In China, approximately 400 million individuals suffer from varying degrees of DED, meaning nearly two in every five people are affected, highlighting the severe public health challenge posed by this condition [1].

The impact of DED extends beyond simple physical discomfort, imposing a massive socioeconomic burden. In the United States, the direct annual management cost per patient is roughly $783, while the total societal cost—accounting for lost productivity and healthcare resources—is estimated at $55 billion [3]. Patients frequently report symptoms such as burning, foreign body sensation, blurred vision, and photophobia, which restrict daily activities like reading, driving, and computer use [4]. Furthermore, DED is strongly linked to mental health, with symptomatic patients showing 1.47 times higher odds of suicidal ideation, and the health utility values for severe DED are comparable to those for severe angina or dialysis [5].

Pathophysiologically, DED is defined by the Tear Film and Ocular Surface Society (TFOS DEWS II) as a multifactorial disease characterized by a loss of tear film homeostasis, accompanied by ocular symptoms, where tear film instability, hyperosmolarity, ocular surface inflammation, and neurosensory abnormalities play etiological roles [6]. This process is often described as a “vicious cycle of inflammation” [6,7]. Initial triggers, such as environmental stress or aqueous deficiency, lead to tear hyperosmolarity, which activates stress-signaling pathways (e.g., MAPK and NF-κB) in corneal epithelial and resident immune cells [6]. These pathways stimulate the release of pro-inflammatory cytokines like IL-1β and TNF-α, and matrix metalloproteinases (MMPs), particularly MMP-9 [6]. These mediators recruit and activate T lymphocytes, creating a self-perpetuating immune response that leads to goblet cell loss and corneal barrier disruption [6,7].

Despite the high prevalence and severity of DED, current clinical management remains suboptimal. Conventional therapies follow a tiered approach, starting with lubricants, warm compresses, and environmental modifications. However, these are often palliative and fail to halt the underlying inflammatory cascade [6,8]. For moderate-to-severe cases, local anti-inflammatory agents such as corticosteroids, Cyclosporine A (CsA), and Lifitegrast are utilized. While corticosteroids provide rapid relief, their long-term use is restricted by risks of intraocular pressure elevation and cataract formation [8]. CsA (e.g., Restasis) is the clinical standard for immunomodulation, yet it is notorious for its slow onset of action, often requiring 3 to 6 months for noticeable improvement [9]. Moreover, its poor aqueous solubility and macrocyclic structure often cause stinging and burning upon instillation, leading to low patient compliance [9,10]. Lifitegrast (Xiidra) has a faster onset (approximately 2 weeks) but is associated with side effects such as dysgeusia (taste disturbance) and local irritation [11]. Consequently, there is an urgent need for novel drugs with superior pharmacological properties and multi-target efficacy.

Teriflunomide, a small-molecule immunomodulator originally approved for relapsing–remitting multiple sclerosis (RRMS) [12,13], has recently shown promising therapeutic potential in dry eye disease (DED) [14]. Its primary mechanism involves the selective and reversible inhibition of mitochondrial dihydroorotate dehydrogenase (DHODH), thereby blocking de novo pyrimidine synthesis and suppressing the proliferation of activated lymphocytes [15]. Notably, in our previous work (bioRxiv preprint) [14], we demonstrated that topical application of Teriflunomide in a benzalkonium chloride (BAC)-induced mouse model of DED significantly alleviated disease phenotypes [16], including reduced corneal opacity, enhanced epithelial healing, and restoration of goblet cell density, with efficacy comparable to the standard-of-care drug loteprednol etabonate [14,17].

However, while phenotypic experiments have confirmed its protective effects, the specific molecular mechanisms and direct therapeutic targets of Teriflunomide on the ocular surface remain largely a “black box” [18]. Given the complexity of the ocular surface microenvironment, explaining its efficacy solely through DHODH inhibition is likely insufficient [6,18]. Parsing the precise targets and pathways of Teriflunomide is not only vital for understanding its pharmacology and optimizing formulations but also essential for the advancement of precision medicine in DED [7,19]. Identifying core targets can provide biomarkers for predicting therapeutic response in a highly heterogeneous patient population [20].

In this study, we implemented an integrated and systematic workflow combining computational pharmacology with multi-scale transcriptomic analysis to elucidate the mechanisms of Teriflunomide in DED. We first evaluated the druggability and ADMET properties of Teriflunomide using ADMETlab 3.0, benchmarking its pharmacokinetic profile against established clinical agents [21]. We then integrated seven independent mouse transcriptomic datasets spanning five distinct induction mechanisms—including genetic defects, immune dysregulation, pharmacological inhibition, chemical injury, and spontaneous autoimmunity—to identify core disease-associated drivers [6,7]. Utilizing single-cell RNA sequencing (scRNA-seq), we further resolved the cell-type-specific expression patterns of these candidate targets at single-cell resolution [22]. Following multi-level target prioritization based on integrated bulk and single-cell transcriptomic analyses, molecular docking was employed as an initial filtering strategy to evaluate the static spatial affinity and binding poses between Teriflunomide and candidate targets. To overcome the limitations of rigid docking, subsequent 50 ns molecular dynamics (MD) simulations were performed to further refine these candidates by assessing the dynamic stability, conformational flexibility, and thermodynamic favorability of the protein–ligand complexes under simulated physiological conditions [23,24]. Subsequently, the selected core targets were subjected to validation using the virtual cell simulation framework AetherCell, which enabled systematic assessment of the functional concordance between target knockdown perturbations and Teriflunomide-induced cellular responses. This approach not only quantified the association between each candidate target and the drug but also provided insights into how individual targets may influence the pathological processes of dry eye disease. This integrated “computation-to-validation” paradigm provides a robust framework for supporting the clinical translation of Teriflunomide as a multi-target therapeutic strategy for DED.

## 2. Materials and Methods

### 2.1. ADMET Profiling and Comparative Analysis of Teriflunomide

To evaluate the pharmacokinetic potential of Teriflunomide, we employed the ADMETlab 3.0 platform to predict its physicochemical properties and ADMET (Absorption, Distribution, Metabolism, Excretion, and Toxicity) profiles [21]. For comparative analysis, three clinically established treatments for dry eye disease (DED)—Cyclosporine, Lifitegrast, and Loteprednol—were included as reference compounds. The analysis integrated several critical molecular descriptors: Molecular Weight (MW), Partition Coefficient (logP), Topological Polar Surface Area (TPSA), Caco-2 Permeability (Caco-2), Aqueous Solubility (logS), Number of Hydrogen Bond Acceptors (nHA), Number of Hydrogen Bond Donors (nHD), and Number of Rotatable Bonds (nRot). These parameters were utilized to comprehensively assess the bioavailability and local ocular surface permeability of the compounds.

### 2.2. Acquisition and Processing of Transcriptomic Data from Dry Eye Mouse Models

Transcriptomic (RNA-seq) datasets related to dry eye disease (DED) in mice were systematically retrieved from the Gene Expression Omnibus (GEO) database [25]. A total of seven datasets representing five distinct induction methods were included: AQP5 knockout model (GSE149832), AIRE knockout models (GSE208297, GSE272701), scopolamine-induced models (GSE186015, GSE316045), benzalkonium chloride (BAC)-induced model (GSE252984), and the NOD spontaneous DED model (GSE306385). Differential expression analysis (DEA) was performed using the DESeq2 package in R (version 4.0) [26]. To ensure data quality, low-abundance genes were filtered out prior to analysis; only genes with a count ≥ 10 in either the treatment or control group (intersection/union method) were retained. Size factors were estimated for normalization. The log2 Fold Change (log_2_FC) values were further refined using the apeglm shrinkage estimator to minimize the impact of noise from low-count genes on the fold-change estimates [27]. *p*-values were adjusted for multiple testing using the Benjamini–Hochberg (BH) procedure to control the false discovery rate (FDR). In this study, genes with |log_2_FC| > 1 and an adjusted *p* value < 0.05 were identified as significantly differentially expressed genes (DEGs). Following DEA, a ranked list of genes based on their log_2_FC was used as input for the fgsea package [28]. Enrichment analysis was conducted against the Gene Ontology Biological Process (GOBP) and Kyoto Encyclopedia of Genes and Genomes (KEGG) databases [29,30]. Pathways with a *p* value < 0.05 were considered significantly enriched and retained for comparative analysis across the datasets.

### 2.3. Identification of Core Leading-Edge Genes

To identify robust biological signatures across different dry eye models, GSEA results from the seven datasets were integrated. Consensus pathways were defined as those significantly enriched (*p* < 0.05) with a consistent direction of regulation (either up- or down-regulated) in at least 50% of the datasets (i.e., ≥4 datasets). The leading-edge genes, which represent the core subset of genes contributing most significantly to the Enrichment Score (ES) and driving the pathway’s biological phenotype, were extracted for each consensus pathway across the contributing datasets. To further narrow down the key drivers, we calculated the occurrence frequency of each leading-edge gene within the datasets where the corresponding pathway was significantly changed. Genes with an occurrence frequency > 0.5 (appearing in more than 50% of the relevant datasets) were identified as “Core Leading-edge Genes.” These genes were prioritized for subsequent molecular network construction and key node analysis.

### 2.4. Identification and Systematic Analysis of Key Genes

Potential therapeutic targets of Teriflunomide were predicted using the SuperPred server via reverse molecular docking [31,32], yielding 113 candidate targets (Table S2). These targets were then intersected with the 885 core leading-edge genes identified in Section 2.2. This integrative approach resulted in 16 key genes that function both as potential pharmacological targets and as core molecular drivers across multiple DED models. The log_2_FC values of these 16 key genes were extracted and compared across the seven transcriptomic datasets to assess their expression consistency. Subsequently, Gene Ontology (GO) enrichment analysis was performed using the clusterProfiler R package to elucidate the biological processes potentially modulated by Teriflunomide in the context of DED [33]. To explore the functional coordination among these key genes, a PPI network was constructed [34]. This analysis aimed to visualize the interaction landscape and identify central hubs within the molecular network, providing insights into the multi-target regulatory mechanism of Teriflunomide.

### 2.5. Acquisition and Processing of Single-Cell Transcriptome Data

Single-cell RNA-sequencing (scRNA-seq) data were obtained from the GEO database (GSE182582). The Cell Ranger (v6.0.1) pipeline was utilized to align reads against the mm10 mouse reference genome and generate filtered feature-barcode matrices [35]. The resulting matrices were imported into Seurat (v4.0) for sample merging, quality control, and downstream analysis [36]. Data were log-normalized and scaled using built-in Seurat functions. After performing Principal Component Analysis (PCA), the Harmony (v1.0) algorithm was applied to integrate samples and mitigate batch effects [37]. UMAP visualization was subsequently conducted based on the Harmony-corrected reductions (dims = 1:22) [38]. Cell clusters were identified using the Louvain algorithm with a resolution of 0.8 [39]. A total of 11 cell subpopulations were annotated: Limbal Stem Cells, Adaptive Limbal Stem Cells, Transit Amplifying Cells, Adaptive Transit Amplifying Cells, Basal Cells, Wing Cells, Adaptive Wing Cells, Squamous Cells, Adaptive Squamous Cells, Conjunctival Epithelial Cells, and Immune Cells. The 16 identified key genes were defined as a functional gene module. The expression scores of this module across various cell subpopulations were calculated using Seurat’s scoring functionality. Furthermore, the differential expression patterns of these 16 key genes were evaluated across different cell types by comparing the dry eye and control groups.

### 2.6. Molecular Docking of Core Target Genes with Teriflunomide

To investigate the structural basis of the interaction between Teriflunomide and the identified core driver genes, molecular docking simulations were performed using AutoDock Vina (version 1.2.7) [23]. The three-dimensional protein structures of six core targets (CTSS, STAT1, PTGS1, CDK1, PSMB9, and TOP2A) were retrieved from the Protein Data Bank (PDB) [40], while the 3D structure of Teriflunomide was obtained from the PubChem database [41]. The docking grid box center and dimensions were defined using a dual strategy: for proteins with co-crystallized ligands, the grid was centered on the native ligand and expanded to cover the validated binding pocket [42]; for proteins without known binding sites, a semi-blind docking approach was applied by defining the grid to cover the major regions of the protein structure [43]. An exhaustiveness value of 32 was applied to ensure a comprehensive search of the conformational space. The docking results were evaluated based on binding affinity, and gene–drug complexes with a binding energy < −6.5 kcal/mol were prioritized for subsequent molecular dynamics (MD) simulations. This threshold was determined based on previous studies, where binding energies ≤ −7 kcal/mol are generally considered strong interactions, while values between −6 and −6.5 kcal/mol represent a transitional range. Therefore, −6.5 kcal/mol is commonly used as a moderate and practical cutoff to balance sensitivity and specificity in virtual screening [44].

### 2.7. Molecular Dynamics Simulation of Core Gene–Teriflunomide Complexes

To evaluate the dynamic stability and binding affinity of Teriflunomide with core target proteins (e.g., STAT1) under physiological conditions, all-atom molecular dynamics (MD) simulations were performed for 50 ns using GROMACS [24]. The optimal docking conformations were converted to Mol2 format via Open Babel [45], and ligand topologies were generated using ACPYPE based on the General Amber Force Field (GAFF) [46,47]. The AMBER99SB-ILDN force field and TIP3P water model were applied for the protein and solvent, respectively [48,49]. The protein-ligand complex was centered in a cubic box with a minimum distance of 1.2 nm from the box edges and solvated with water. Counterions (Na^+^/Cl^−^) were added to neutralize the system’s net charge. The equilibration procedure consisted of: (1) Energy minimization using the steepest descent algorithm; (2) A 100 ps NVT (constant number of particles, volume, and temperature) equilibration at 310 K using the V-rescale thermostat; and (3) A 100 ps NPT (constant number of particles, pressure, and temperature) equilibration at 1 atm using the Parrinello-Rahman barostat [50,51]. Position restraints were applied to the protein backbone and ligand during these stages. A 50 ns production MD simulation was subsequently performed without restraints. The LINCS algorithm was used to constrain all hydrogen bonds [52], with an integration time step of 2 fs. Non-bonded interactions were truncated at 1.0 nm, and long-range electrostatic interactions were handled using the Particle Mesh Ewald (PME) method [53]. To optimize computational performance, simulations were executed on a high-performance computing platform with GPU acceleration. Post-simulation analyses, including Root Mean Square Deviation (RMSD), Root Mean Square Fluctuation (RMSF), and Radius of Gyration (Rg), were conducted to assess the structural stability of the complexes.

### 2.8. Simulation of Key Gene–Teriflunomide Relationships via AetherCell Virtual Cell Model

**Figure 1:**
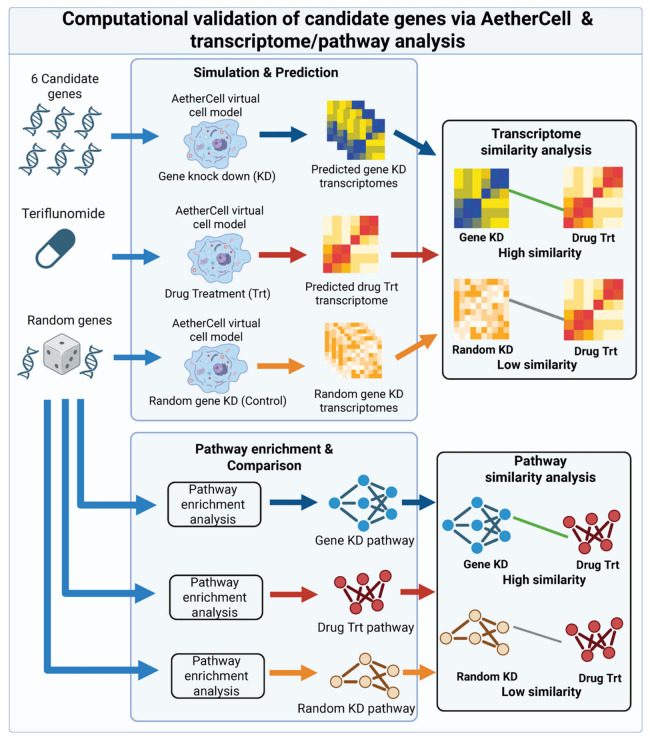
Computational workflow for key gene–teriflunomide relationships via AetherCell virtual cell model.

The regulatory relationship between key genes and Teriflunomide was systematically simulated using the self-developed virtual cell engine, AetherCell [14]. The seven mouse transcriptomic datasets described in Section 2.1 were employed as baseline expression matrices for the model. We simulated the transcriptomic perturbations induced by the knockdown (KD) of individual key genes as well as those induced by Teriflunomide treatment. To quantify the association between these genes and the drug’s mechanism, the Pearson Correlation Coefficient (PCC) was calculated to measure the similarity between the log_2_FC profiles of gene KD and Teriflunomide treatment. A null distribution was generated by simulating the knockdown of 100 randomly selected genes as a control. Furthermore, pathway enrichment analysis was performed on the simulated transcriptomic profiles using the fgsea package. Using the pathway alterations induced by Teriflunomide as the ground truth (label) and the alterations induced by gene KD as the predicted values, the Area Under the Receiver Operating Characteristic curve (AUROC) was calculated to assess the functional similarity of biological perturbations. The same procedure was applied to the 100 random genes to serve as a baseline control, thereby evaluating the specificity and significance of the identified key genes in mediating the pharmacological effects of Teriflunomide. The overall simulation workflow is illustrated in Figure 1.

## 3. Results

### 3.1. Integrated Transcriptomic Landscape and Identification of Consensus Pathological Drivers in DED

**Figure 2:**
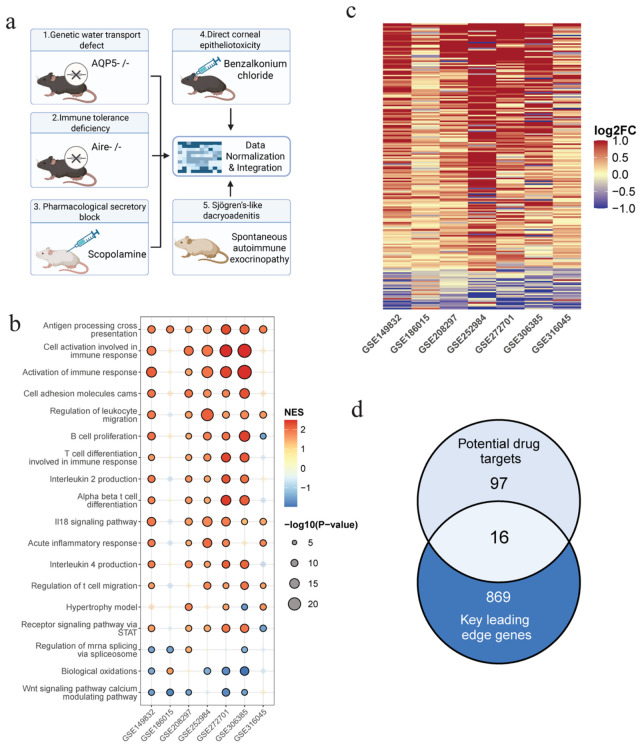
Cross-model transcriptomic integration reveals consensus pathological pathways and core candidate genes in dry eye disease. (**a**) Schematic overview of the five distinct dry eye disease (DED) induction strategies integrated in this study. (**b**) Consensus bubble plot of Gene Set Enrichment Analysis (GSEA) results across seven independent transcriptomic datasets. Bubble color indicates the normalized enrichment score (NES), with red representing pathway activation and blue representing pathway inhibition. Bubble size corresponds to −log10 (*p*-value). (**c**) Heatmap showing the expression patterns of key leading-edge genes across the seven datasets. The color scale represents log2 fold change (log_2_FC), with red indicating up-regulation and blue indicating down-regulation. (**d**) Venn diagram illustrating the overlap between predicted Teriflunomide drug targets and conserved key leading-edge genes.

To comprehensively characterize the shared pathological features of dry eye disease (DED) across heterogeneous experimental settings, we integrated seven mouse transcriptomic datasets representing five distinct induction mechanisms, including genetic water transport defect (AQP5^−/−^), immune tolerance deficiency (Aire^−/−^), pharmacological secretory block (scopolamine-induced), direct corneal epitheliotoxicity (benzalkonium chloride-induced), and Sjögren’s-like dacryoadenitis (NOD model) (Figure 2a). The comparative ADMET profiling of Teriflunomide and the dataset-specific differential expression landscapes are presented in the Supplementary Materials (Supplementary Figures S1 and S4). In addition, principal component analysis and inter-sample correlation heatmaps further confirmed satisfactory sample clustering and within-group consistency across datasets (Supplementary Figures S2 and S3), supporting the robustness of the subsequent integrative analysis.

Meta-analysis of GSEA results revealed highly conserved transcriptional programs across the seven datasets (Figure 2b). Multiple immune- and inflammation-related pathways were recurrently enriched, most notably Antigen processing and cross-presentation, Cell activation involved in immune response, Activation of immune response, and STAT-mediated receptor signaling, all of which showed consistent up-regulation across most models. In contrast, pathways such as Biological oxidations and Wnt signaling pathway calcium modulating pathway exhibited a relatively stable down-regulation trend. These findings indicate that, despite the marked heterogeneity in model induction strategies, DED pathology converges on a shared inflammatory and immune-regulatory axis. Consistently, leading-edge gene analysis further demonstrated highly synchronized expression patterns across all seven datasets, with clear clusters of both up-regulated and down-regulated genes (Figure 2c), reinforcing the reliability of these genes as conserved molecular signatures of DED.

To further identify therapeutically relevant targets, we intersected the predicted targets of Teriflunomide with the conserved leading-edge genes. As shown in Figure 2d, the overlap analysis identified 16 shared genes between the 97 potential drug targets and the 869 key leading-edge genes (Table S1). These 16 genes were considered both potential pharmacological targets of Teriflunomide and central molecular drivers across multiple DED pathological contexts, and were therefore prioritized for downstream functional analysis and single-cell validation.

### 3.2. Functional Enrichment and Interaction Network Analysis of Key Candidate Genes

**Figure 3:**
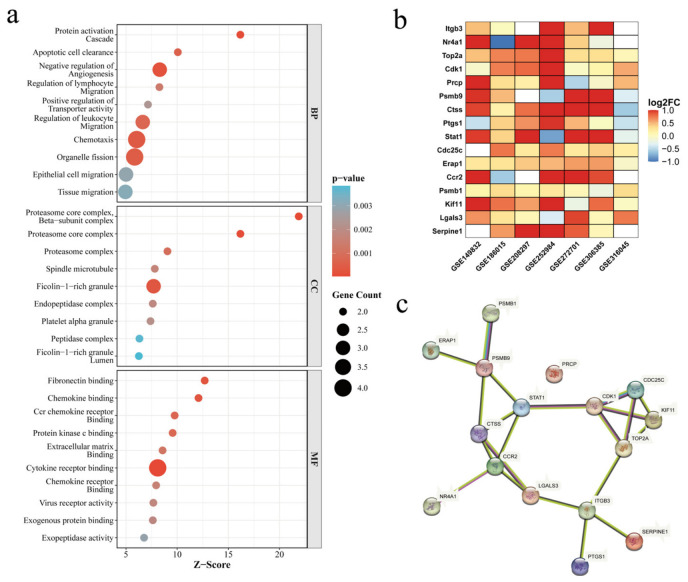
Functional characterization and interaction analysis of the 16 core candidate genes. (**a**) Gene Ontology (GO) enrichment analysis of the 16 core leading edge genes, categorized into Biological Process (BP), Cellular Component (CC), and Molecular Function (MF). Bubble color represents the *p*-value, and bubble size indicates the gene count. The *x*-axis denotes the Z-score. (**b**) Heatmap showing the log_2_FC of the 16 core candidate genes across seven independent datasets. (**c**) Protein–protein interaction (PPI) network of the 16 core candidate genes. Nodes represent proteins, and edges represent documented interactions, highlighting the central roles of STAT1, CTSS, and other hub genes within the network.

To elucidate the biological functions of the 16 key genes, Gene Ontology (GO) enrichment analysis was performed (Figure 3a). In the Biological Process (BP) category, these genes were significantly enriched in protein activation cascades, apoptotic cell clearance, regulation of lymphocyte migration, and chemotaxis. Regarding Cellular Component (CC), they were primarily localized to proteasome complexes and Ficolin-1-rich granules. In terms of Molecular Function (MF), the genes exhibited strong activities in chemokine binding, cytokine receptor binding, and fibronectin binding. These findings suggest that the 16 candidate genes modulate the immuno-inflammatory cascade in DED across multiple functional dimensions.

The heatmap (Figure 3b) illustrates the differential expression profiles of the 16 key genes across the seven datasets. Most genes, including Ctss, Stat1, Serpine1, Top2a, and Cdc25c, exhibited robust up-regulation patterns across all models, with particularly high fold changes observed in the AQP5-knockout (GSE149832), BAC-induced (GSE252984) and Aire-knockout (GSE272701) datasets. This consistent expression across diverse models reinforces their potential as universal pathological drivers of DED.

To elucidate the functional relationships among these 16 potential targets, a Protein–Protein Interaction (PPI) network was constructed using the STRING database (Figure 3c). The topology of this network reveals a remarkably high degree of connectivity among the candidates. Notably, rather than acting in isolation, these 16 genes form a tightly integrated functional cluster with multiple reciprocal interactions. This high level of network interdependency suggests that Teriflunomide may achieve its therapeutic efficacy through a coordinated, systemic perturbation of this core regulatory module, rather than by modulating independent and separate targets.

### 3.3. Single-Cell Profiling Reveals Cell-Type-Specific Expression of Key Genes

**Figure 4:**
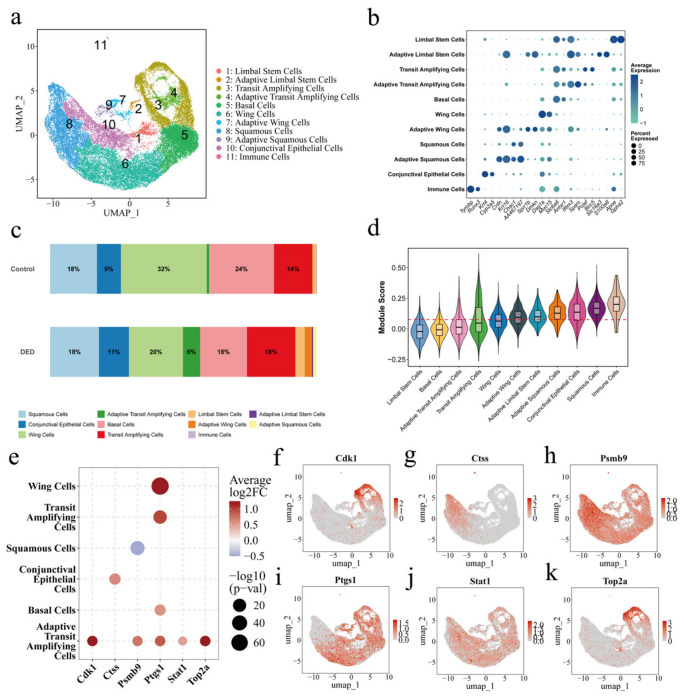
Single-cell RNA-seq reveals corneal epithelial heterogeneity and candidate gene expression profiles in DED. (**a**) UMAP visualization of corneal epithelial and immune cells, identifying 11 distinct cell clusters. (**b**) Dot plot showing the expression of representative marker genes for each cell cluster. (**c**) Bar chart comparing the proportional distribution of cell clusters between Control and DED groups. (**d**) Violin plot illustrating the module scores of the 16 core candidate genes across different cell populations. (**e**) Dot plot showing the differential expression (log_2_FC) and significance of six representative candidate genes across major cell types. (**f**–**k**) Feature plots displaying the spatial expression distribution of Cdk1, Ctss, Psmb9, Ptgs1, Stat1, and Top2a within the single-cell landscape.

To resolve the expression patterns of key genes at single-cell resolution, we performed scRNA-seq analysis on ocular surface tissues. UMAP visualization identified 11 distinct cell clusters (Figure 4a), encompassing various corneal epithelial lineages, conjunctival epithelial cells, and immune cells. The cluster-specific expression of marker genes (Figure 4b) confirmed the accuracy of the cell-type annotation.

Analysis of cell proportions revealed a significant expansion of ‘Adaptive’ cell populations in the DED group, which we identified as the activated pathological states of the ocular surface. Specifically, the marked increase in Adaptive Wing, Limbal Stem, and Squamous cells, accompanied by a reduction in quiescent Wing (32% to 20%) and Basal (24% to 18%) cells (Figure 4c), reflects extensive epithelial remodeling and a shift toward a stress-responsive state under DED conditions. Evaluation via Module Scores further showed that the 16 candidate genes were predominantly enriched in these activated adaptive clusters and immune cell populations (Figure 4d), identifying them as the primary functional hubs of DED pathology.

To pinpoint the most robust effectors, we analyzed the expression profiles of the 16 candidates across major subclusters (Figure 4e). Six genes—Cdk1, Ctss, Psmb9, Ptgs1, Stat1, and Top2a—emerged as the core drivers. Notably, Ptgs1 showed the most extensive up-regulation across nearly all epithelial lineages. In contrast, Cdk1 and Top2a exhibited highly synchronized induction specifically within the Adaptive Transit Amplifying Cells, suggesting altered cell cycle kinetics in this pathological state. Furthermore, Ctss and Stat1 were primarily elevated in Conjunctival Epithelial Cells, highlighting a localized immune-regulatory response. The UMAP feature plots (Figure 4f–k) confirmed these spatial expression preferences. Consequently, these six genes were prioritized as the core therapeutic targets of Teriflunomide for subsequent virtual cell simulations and molecular docking analysis.

### 3.4. Molecular Docking Simulation of Teriflunomide with Core Target Proteins

**Figure 5:**
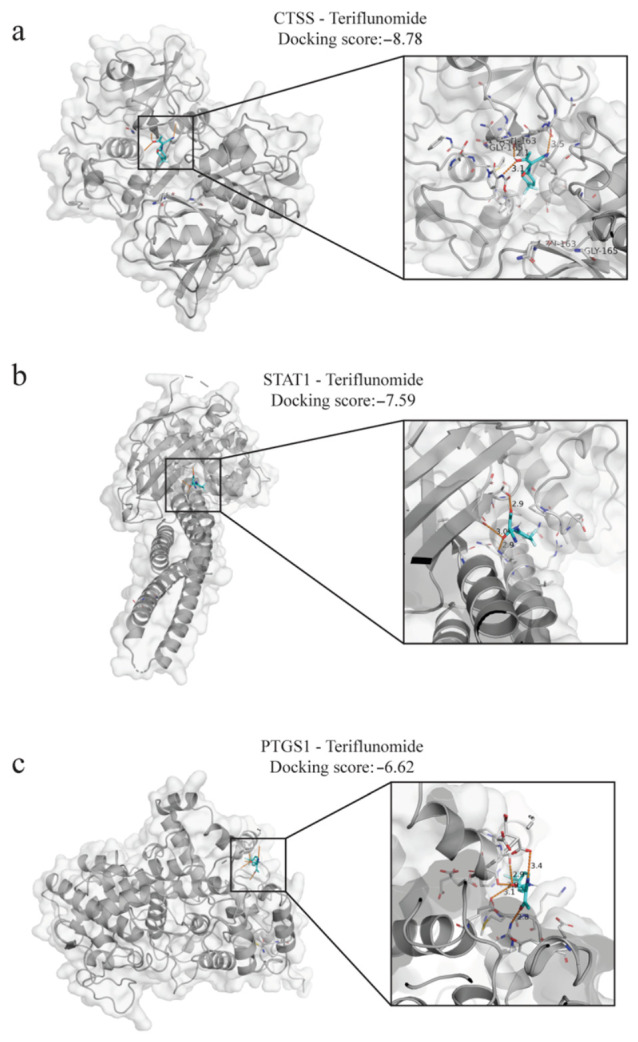
Molecular docking modes and interaction stability of Teriflunomide with core target proteins. (**a**–**c**) Molecular docking conformations of Teriflunomide with CTSS (**a**), STAT1 (**b**), and PTGS1 (**c**). The left panels show the overall view of the protein surface and ligand-binding pockets; the right enlarged panels illustrate specific interactions between the ligand and pocket residues. Key hydrogen bonds (distances in Å) and docking scores are indicated. Proteins are shown as gray cartoon/surface representations, Teriflunomide is displayed as cyan sticks, and interacting amino acid residues are shown as gray sticks. Orange dashed lines represent hydrogen bonds.

To further explore the direct interactions between Teriflunomide and the core targets, molecular docking simulations were performed on the protein products of the six core genes identified through single-cell validation (Table 1). Based on a binding energy threshold of <−6.5 kcal/mol, three high-affinity complexes were identified: CTSS-Teriflunomide (−8.78), STAT1-Teriflunomide (−7.59), and PTGS1-Teriflunomide (−6.62) (Figure 5a–c). The significantly low binding energy values indicate robust stable physical binding potential between Teriflunomide and these proteins.

Detailed binding mode analysis elucidated the positioning of Teriflunomide within the active pockets of the proteins: CTSS-Teriflunomide (Figure 5a): Teriflunomide is stably embedded within the catalytic pocket of CTSS, forming prominent hydrogen bonds with residues such as GLY-165 (bond distances of 2.1 Å, 3.1 Å and 3.5 Å, exhibiting the strongest binding stability among the tested targets. STAT1-Teriflunomide (Figure 5b): Teriflunomide occupies a critical domain of STAT1, interacting closely with surrounding residues via multiple hydrogen bonds, achieving a docking score of −7.59. This suggests a potential direct interference with the STAT1 signaling transduction process. PTGS1-Teriflunomide (Figure 5c): In the PTGS1 model, Teriflunomide interacts through a complex hydrogen-bond network with specific residues (bond distances ranging from 2.8 Å to 3.4 Å), providing a structural rationale for its inhibition of prostaglandin synthesis.

In summary, the molecular docking results validate the binding capacity of Teriflunomide toward CTSS, STAT1, and PTGS1 at the atomic level. The exceptional affinity observed for CTSS and STAT1 positions them as primary candidate targets for elucidating the molecular mechanism of Teriflunomide in treating DED.

### 3.5. Molecular Dynamics Simulation and Free Energy Landscape Analysis Support Stable Target Engagement of Teriflunomide

**Figure 6:**
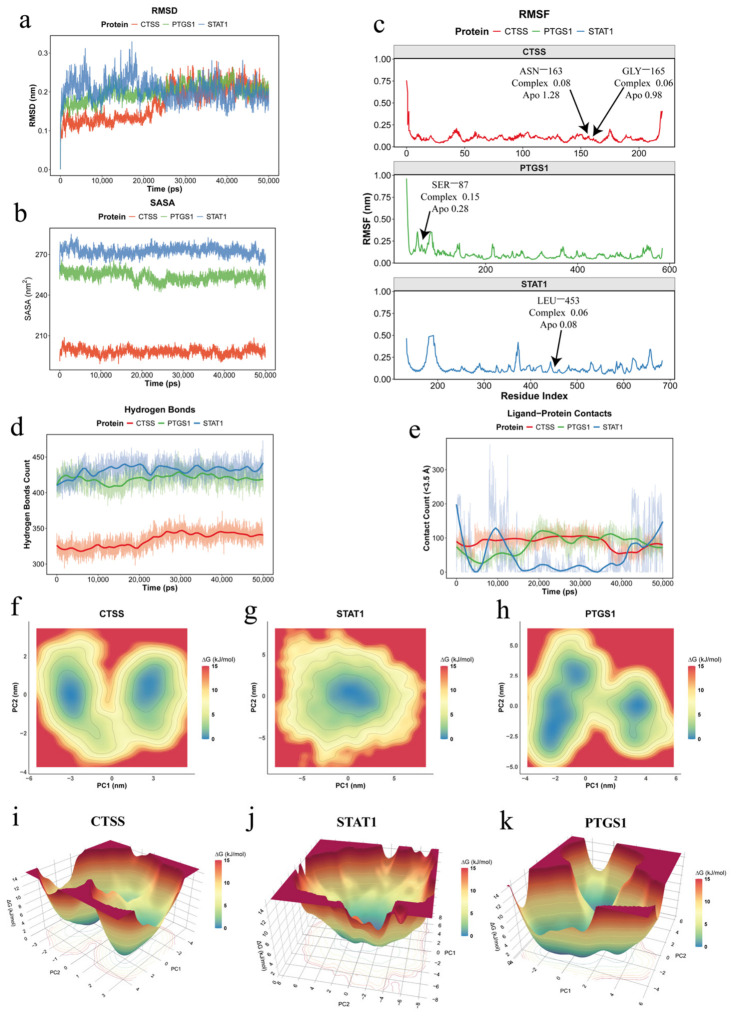
Molecular dynamics simulation and free energy landscape analysis of Teriflunomide in complex with core target proteins. (**a**) Root Mean Square Deviation (RMSD) showing the time-dependent displacement of protein backbone atoms relative to the initial conformation. (**b**) Solvent Accessible Surface Area (SASA) representing the protein surface area exposed to solvent during the simulation. (**c**) Root Mean Square Fluctuation (RMSF) illustrating residue-level flexibility relative to the average structure, with the *x*-axis indicating residue indices. Key residues involved in ligand interactions are highlighted, with annotations indicating their RMSF values in both apo and complex states. (**d**) Hydrogen bond analysis showing the dynamic changes in the number of hydrogen bonds formed during the molecular dynamics simulation. Light-colored lines represent raw hydrogen bond counts, while dark lines indicate the smoothed trend over time. (**e**) Ligand–protein contact count (<3.5 Å) quantifying the number of atom pairs between Teriflunomide and each protein within a 3.5 Å distance; light-colored lines represent raw values and dark lines indicate the moving average. (**f**–**h**) Two-dimensional (2D) free energy landscape (FEL) projections of the CTSS, STAT1, and PTGS1 complexes, respectively, based on the first two principal components (PC1 and PC2). (**i**–**k**) Corresponding three-dimensional (3D) representations of the FEL for CTSS, STAT1, and PTGS1, respectively. The color scale indicates the free energy value (ΔG, kJ/mol), ranging from blue (lowest energy) to red (highest energy). Deep blue regions represent the most populated and thermodynamically stable conformational states during the simulation.

To further evaluate the dynamic stability of Teriflunomide in complex with the three core targets (CTSS, PTGS1, and STAT1), 50 ns molecular dynamics (MD) simulations were performed (Figure 6a–e). The RMSD profiles showed that all three systems rapidly reached equilibrium and remained overall stable throughout the simulation (Figure 6a). Among them, CTSS and PTGS1 displayed relatively smaller fluctuations, whereas STAT1 exhibited broader conformational adjustments within the range of approximately 0.2–0.3 nm, which is likely attributable to its larger molecular size and intrinsic structural flexibility. Consistently, SASA analysis demonstrated stable solvent-accessible surface areas across all complexes, indicating that ligand binding did not disrupt the compact folding state of the proteins (Figure 6b).

Notably, comparable analyses of the apo systems revealed generally similar stability profiles but with relatively higher residue-level fluctuations, supporting the notion that ligand binding contributes to local structural stabilization (Supplementary Figure S5).

At the residue level, RMSF analysis revealed that fluctuations were mainly localized to flexible loop and linker regions (Figure 6c). Notably, key residues involved in ligand interactions exhibited markedly reduced fluctuations in the complex state compared to the apo state (e.g., GLY-165 and ASN-163 in CTSS, SER-87 in PTGS1, and LEU-453 in STAT1), indicating ligand-induced local stabilization. STAT1 showed relatively increased flexibility at several domain-connecting regions, consistent with its multidomain signaling architecture and conformational plasticity. In addition, all three complexes maintained stable hydrogen-bonding networks throughout the simulation (Figure 6d), supporting persistent ligand–protein association. The ligand–protein contact analysis further revealed distinct interaction patterns among the three complexes (Figure 6e). CTSS and PTGS1 exhibited relatively stable and sustained short-range contacts, whereas the STAT1 complex showed more dynamic fluctuations in contact density. Importantly, this fluctuating contact profile did not indicate dissociation, as the sustained hydrogen bonds suggest that Teriflunomide remained anchored within a flexible binding pocket and transitioned between multiple semi-stable microstates. Collectively, these MD results support the formation of dynamically stable complexes between Teriflunomide and all three core targets.

To further characterize the thermodynamic conformational landscape of these complexes, free energy landscape (FEL) analysis was performed based on the first two principal components (PC1 and PC2) (Figure 6f–k). Here, PC1 and PC2 capture the dominant collective motions of the systems, including global breathing, inter-domain shearing, and localized loop fluctuations, depending on the protein context (Supplementary Figure S6). The 2D and 3D FEL plots consistently showed well-defined low-energy basins, indicating thermodynamically favorable conformational states. Specifically, the CTSS complex displayed a clear bi-basin distribution with a relatively low energy barrier between the two major minima (Figure 6f,i), suggesting flexible transitions between two stable conformational states. In contrast, STAT1 exhibited a broad central energy basin (Figure 6g,j), indicating convergence toward a dominant low-energy conformational space despite its larger dynamic fluctuations observed in MD analysis. The PTGS1 complex showed multiple dispersed low-energy sub-basins (Figure 6h,k), reflecting a more complex conformational landscape and possible multi-stage structural fine-tuning during ligand binding.

Overall, the combined MD and FEL analyses demonstrate that Teriflunomide can stably and spontaneously engage CTSS, STAT1, and PTGS1 at the biophysical level. These findings provide strong mechanistic support for the multi-target therapeutic action of Teriflunomide in dry eye disease.

### 3.6. Virtual Cell-Based Validation Links Target Perturbation to Teriflunomide-Induced Transcriptional and Pathway Responses

**Figure 7:**
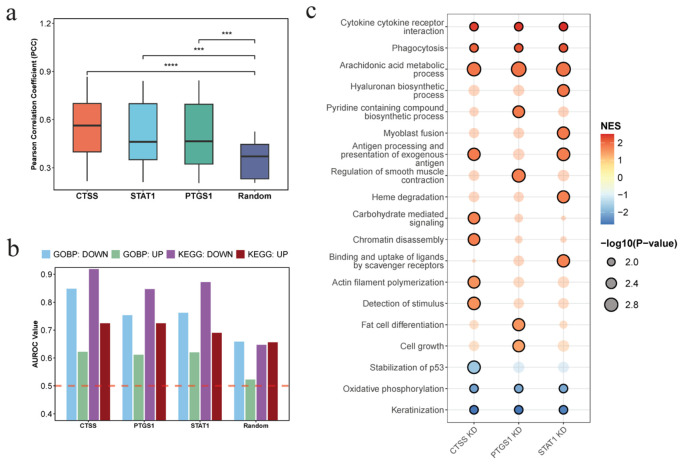
AetherCell-based correlation analysis between virtual gene knockdown and Teriflunomide treatment. (**a**) Box plot showing the Pearson Correlation Coefficient (PCC) between the predicted expression profiles of individual gene knockdowns and the predicted profile of Teriflunomide treatment. *p*-values were calculated using the unpaired Wilcoxon rank-sum test (*** *p* < 0.001, **** *p* < 0.0001). (**b**) Bar chart illustrating the AUROC values for GO-BP and KEGG gene sets, using Teriflunomide-induced pathway changes as the ground truth (Label) and gene knockdown-induced changes as the prediction (Predict). The red dashed line indicates AUROC = 0.5, representing the performance expected from random classification. (**c**) Heatmap of differential functional enrichment among the 3 core candidates.

Following molecular docking and molecular dynamics simulations, CTSS, STAT1, and PTGS1 were identified as candidate targets of teriflunomide for dry eye disease (DED). To further validate their functional relevance, we leveraged the AetherCell virtual cell model to simulate gene perturbation and compare it with teriflunomide-induced cellular responses. At the transcriptomic level, Pearson correlation coefficient (PCC) analysis based on the top 20% most altered genes revealed that all three candidate targets showed substantially higher concordance with teriflunomide-induced expression profiles than the random control (Figure 7a). Among them, CTSS exhibited the strongest correlation, followed by STAT1 and PTGS1, indicating that perturbation of these targets can effectively recapitulate drug-induced transcriptional changes.

At the pathway level, AUROC analysis demonstrated that gene perturbations of CTSS, STAT1, and PTGS1 achieved consistently higher predictive performance than random controls in most pathway categories (Figure 7b). Notably, KEGG downregulated pathways and GO-based pathways showed clear separation from the random baseline, supporting the biological relevance of these targets. In contrast, KEGG upregulated pathways exhibited relatively limited discrimination from random perturbations, suggesting that teriflunomide-induced pathway activation may involve more complex or indirect regulatory mechanisms that are not fully captured by single-gene perturbation.

To further dissect their mechanistic roles, pathway enrichment analysis was performed for each target (Figure 7c). Several shared pathways—including cytokine—cytokine receptor interaction, phagocytosis, and arachidonic acid metabolism—were consistently modulated across all three targets, highlighting a common role in immune regulation and inflammatory response, which are central to DED pathology.

Importantly, each target also exhibited distinct functional specializations, suggesting complementary roles in mediating the therapeutic effects of teriflunomide. CTSS uniquely regulated pathways related to detection of stimulus and actin filament polymerization, indicating its primary role in alleviating ocular surface irritation and restoring epithelial barrier integrity. PTGS1 was specifically associated with pathways such as pyridine-containing compound biosynthetic process and regulation of smooth muscle contraction, suggesting its involvement in modulating lipid metabolism and maintaining microvascular homeostasis at the ocular surface. In contrast, STAT1 emerged as a central regulatory hub, not only suppressing inflammation through cytokine–cytokine receptor interaction, but also uniquely promoting hyaluronan biosynthetic process, implying a dual role in lubrication enhancement and tissue repair.

Collectively, these findings reveal that while CTSS, PTGS1, and STAT1 share a common immunomodulatory framework, they contribute to teriflunomide’s therapeutic effects through distinct yet complementary mechanisms, encompassing inflammation suppression, epithelial barrier restoration, metabolic regulation, and tissue repair.

## 4. Discussion

The current investigation provides a high-resolution molecular and computational roadmap of Teriflunomide’s therapeutic efficacy in dry eye disease (DED), transcending the traditional understanding of its role as a selective inhibitor of mitochondrial dihydroorotate dehydrogenase (DHODH). By leveraging a multi-scale analytical framework—integrating pharmacokinetic profiling, multi-model transcriptomics, single-cell resolution, and virtual cell simulations—this research decodes the “black box” of Teriflunomide’s pharmacology on the ocular surface, identifying a synergistic three-target mechanism involving CTSS, PTGS1, and STAT1.

The study initially established the pharmacological viability of Teriflunomide through exhaustive ADMET profiling (Supplementary Figure S1). Compared to standard-of-care macrocyclic agents like Cyclosporine A, Teriflunomide exhibits a highly balanced small-molecule profile conducive to biological membrane penetration. Its high predicted Caco-2 permeability and low Topological Polar Surface Area (TPSA) suggest an enhanced capacity to traverse the complex, lipid-rich corneal epithelial barrier. This pharmacokinetic advantage is critical for topical ocular delivery, where the ability to reach deep epithelial layers while maintaining stability in the tear film determines clinical success.

The transcriptomic integration component of the study addressed the pathological heterogeneity of DED by combining data from seven independent mouse models. These models represented diverse induction strategies, including genetic water transport defects (AQP5^−/−^), immune tolerance deficiencies (Aire^−/−^), and chemical injury (BAC-induced). Despite the variation in induction methods, the models collectively converged on inflammatory activation and epithelial barrier disruption. The intersection of consensus “leading edge” genes with predicted pharmacological targets yielded 16 core candidates, which were then resolved at single-cell resolution. The scRNA-seq analysis identified 11 distinct cell clusters and revealed a significant expansion of “adaptive” or activated epithelial states under DED conditions, identifying these populations as the primary functional hubs of pathology. Subsequent high-precision molecular docking and 50 ns molecular dynamics (MD) simulations prioritized three targets—CTSS, PTGS1, and STAT1—based on their atomic-level binding stability and thermodynamic convergence.

The identification of Cathepsin S (CTSS) as a primary target of Teriflunomide is particularly significant given its emerging role as a key biomarker and mediator of ocular surface inflammation and neuropathic pain [54]. CTSS is a unique lysosomal cysteine protease that maintains enzymatic stability and activity at neutral or slightly alkaline pH, allowing it to perform both intracellular roles in antigen presentation and extracellular roles in tissue remodeling [55]. Our analysis, supported by the AetherCell virtual cell model and pathway enrichment, indicates that Teriflunomide-mediated inhibition of CTSS addresses two critical clinical features of DED: chronic ocular pain and physical barrier leakage.

Pathologically, CTSS functions as a potent regulator of “detection of stimulus” and nociceptive signaling. Extracellular CTSS has been shown to act as a biased agonist for Protease-Activated Receptor 2 (PAR2), cleaving it at a specific site (E56-T57) that differs from the canonical trypsin cleavage site [55]. This non-canonical activation triggers the release of inflammatory mediators and sensitizes transient receptor potential vanilloid 4 (TRPV4) on corneal nociceptors, leading to neuronal hyperexcitability [56]. This mechanism explains the persistent burning and foreign body sensations reported by patients, which often correlate poorly with clinical signs of tear volume [57]. By forming multiple stable hydrogen bonds within the catalytic pocket of CTSS (docking score −8.77), Teriflunomide prevents the proteolytic activation of the CTSS/PAR2/TRPV4 axis, effectively providing rapid relief from ocular irritation.

In addition to its role in pain, CTSS is a unique regulator of “actin filament polymerization” and epithelial barrier integrity. The corneal epithelium relies on a well-organized actin cytoskeleton to support tight junction (TJ) proteins like ZO-1 and occludin, which maintain the ocular surface’s physical barrier [58]. Research indicates that excessive CTSS levels, induced by hyperosmolarity or proinflammatory cytokines (e.g., TNF-α, IFN-γ), lead to the disruption of these junctional proteins and loss of barrier function [59]. CTSS promotes pathological “epithelial actin reorganization,” which destabilizes the cellular architecture. Teriflunomide’s ability to inhibit CTSS ensures the maintenance of compact protein folding and actin polymerization, thereby reinforcing the physical barrier and preventing the infiltration of noxious stimuli and inflammatory cells.

The second key target, Prostaglandin-Endoperoxide Synthase 1 (PTGS1, also known as COX-1), may reflect Teriflunomide’s capacity to modulate the evaporative and metabolic components of DED. While PTGS2 is more commonly emphasized in acute inflammatory responses, PTGS1 is constitutively expressed and plays an essential role in maintaining physiological homeostasis, including microvascular tone and mucosal protection [60]. Importantly, direct therapeutic evidence for PTGS1 in dry eye disease remains relatively scarce, making it less established than CTSS or STAT1 in the current DED literature. At the same time, this relative underrepresentation may indicate that PTGS1 constitutes a potentially novel therapeutic target in DED rather than merely a secondary inflammatory mediator. In our study, PTGS1 was linked to pathways such as pyridine metabolism and smooth muscle contraction, suggesting a possible role in metabolic regulation and ocular surface homeostasis. These findings position PTGS1 as an exploratory but potentially important target that broadens current understanding of Teriflunomide’s mechanism of action and expands the therapeutic landscape of dry eye disease.

The modulation of “pyridine-containing compound biosynthetic process” by Teriflunomide-PTGS1 interaction is a novel insight into ocular surface metabolism. Pyridine compounds, particularly nicotinamide and its derivatives (NAD^+^), are essential for mitochondrial oxidative phosphorylation and cellular energy balance [61]. Chronic inflammation in DED is often driven by metabolic reprogramming, where oxidative stress and mitochondrial DNA damage exacerbate epithelial injury [62]. By optimizing the biosynthetic processes of these metabolic intermediates, Teriflunomide supports the energetic requirements for epithelial cell migration and repair while mitigating oxidative damage. Furthermore, PTGS1 is involved in regulating the lipid environment of the tear film; its presence in epithelial lineages suggests a role in maintaining the quality of meibum-like secretions that prevent excessive tear evaporation [63].

Additionally, PTGS1 serves as a critical regulator of “smooth muscle contraction” and hemodynamic stability at the ocular surface. Notably, smooth muscle contraction plays a central role in regulating microvascular tone by controlling vasoconstriction and vasodilation, thereby directly influencing local blood flow dynamics. DED patients frequently exhibit conjunctival microvascular alterations, such as increased blood flow velocity (BFV), wall shear rate (WSR), and vessel diameter, which serve as objective indicators of ocular surface inflammation [64]. These changes are often mediated by prostaglandins and other eicosanoids that influence smooth muscle contraction in the conjunctival vasculature [65]. The study’s docking and MD simulations indicate that Teriflunomide forms a stable physical complex with PTGS1, suggesting that it can normalize microvascular responses. By modulating the production of vasoactive lipid mediators, Teriflunomide helps restore microvascular homeostasis, reducing the conjunctival injection (redness) and congestion that characterize severe DED subtypes like Sjögren’s syndrome [66].

STAT1 (Signal Transducer and Activator of Transcription 1) emerged as the core regulatory hub in our multi-target model. While STAT1 is traditionally known for mediating interferon-induced proinflammatory responses, current research reveals its modular signaling architecture and dual role in maintaining epithelial integrity [67]. In the Teriflunomide-treated ocular surface, STAT1 operates through two primary mechanisms: the suppression of “cytokine-cytokine receptor interaction” and the unique activation of “hyaluronan biosynthetic process”.

The inhibition of “cytokine-cytokine receptor interaction” by Teriflunomide via STAT1 provides a powerful break to the “vicious cycle of inflammation”. Initial triggers like hyperosmolarity activate stress-signaling pathways that release IL-1β, TNF-α, and IFN-γ, which in turn activate STAT1 to recruit further immune cells. The AetherCell validation demonstrated that STAT1 perturbation achieved high predictive performance in recapitulating Teriflunomide’s downregulated inflammatory pathways. This target-specific modulation ensures that the self-perpetuating immune response is dampened without inducing the broad immunosuppression associated with systemic steroids.

Perhaps the most profound finding is STAT1’s unique activation of the “hyaluronan biosynthetic process”. Hyaluronan (HA), or hyaluronic acid, is a ubiquitous glycosaminoglycan essential for ocular surface lubrication, tissue hydration, and the maintenance of the limbal stem cell niche [68]. In severe DED, the loss of lubrication leads to increased friction, which damages the corneal epithelium and subbasal nerves [69]. While exogenous HA is a standard component of artificial tears, the promotion of endogenous HA synthesis represents a leap from palliative care to active tissue repair [70]. HA facilitates epithelial wound healing by promoting cell migration and attachment through the CD44 receptor [71]. The enrichment of HA biosynthesis pathways for the STAT1 target suggests that Teriflunomide helps restore the ocular surface’s physical and biological landscape, supporting the regeneration of the epithelial barrier and the health of the corneal stem cell population [72].

The significance of this study lies in resolving the pharmacological complexity of Teriflunomide on the ocular surface. Importantly, the targets prioritized in this work encompass both relatively well-supported DED-associated nodes and more underexplored candidate targets, thereby providing a target landscape with both mechanistic robustness and translational novelty. Specifically, CTSS and STAT1 represent comparatively well-grounded dry eye-related targets that help anchor the mechanistic interpretation of Teriflunomide action, particularly in relation to ocular surface inflammation, epithelial dysfunction, and immune regulation. In contrast, PTGS1 emerges as a comparatively underexplored target in the context of dry eye disease, adding a stronger element of novelty to our findings and expanding the current view of therapeutically actionable pathways. By defining target–pathway relationships, we identify novel therapeutic targets for dry eye disease and provide potential biomarkers for predicting treatment responses in heterogeneous populations. At the same time, this integrated target framework helps distinguish between targets that primarily strengthen the mechanistic plausibility of Teriflunomide and those that broaden the exploratory space for future DED therapeutic development. Moreover, the application of the “computation-to-validation” paradigm, particularly through the AetherCell virtual cell model, establishes a generalizable framework for mechanistic drug analysis and offers a bioinformatics strategy for validating drug repositioning outcomes. Nevertheless, several limitations should be acknowledged. First, the present study is primarily based on integrated transcriptomic analysis, molecular simulations, and virtual-cell-based inference, and therefore still lacks direct experimental validation of target engagement and downstream functional effects. Second, although multiple mouse DED models were integrated to improve robustness, species differences and model-specific biases may limit the direct extrapolation of these findings to human disease. Third, while PTGS1 adds an important layer of novelty, its direct therapeutic relevance in DED remains less established than that of CTSS and STAT1 and will require further validation in dedicated experimental systems. In addition, AetherCell provides an orthogonal and informative in silico validation layer, but its predictions should still be interpreted alongside future in vitro, in vivo, and clinical evidence. Overall, this work supports Teriflunomide as a promising therapeutic option for DED and advances the development of precision medicine in ocular surface diseases.

## 5. Conclusions

This research provides a comprehensive and exhaustive elucidation of the multi-target mechanisms of Teriflunomide in dry eye disease, establishing a new pharmacological paradigm for its application in ophthalmology. By integrating multi-scale transcriptomics, single-cell resolution landscapes, and high-precision virtual cell simulations, the study demonstrates that Teriflunomide’s therapeutic efficacy far exceeds its canonical role as a DHODH inhibitor. The drug acts as a sophisticated immunomodulator that simultaneously addresses the neurosensory, hemodynamic, and epithelial components of DED pathophysiology. The findings pinpoint three primary therapeutic axes: the inhibition of CTSS, which provides rapid relief from ocular pain and restores the physical epithelial barrier by preventing pathological actin remodeling and nociceptor sensitization; the modulation of PTGS1, which optimizes the ocular surface lipid environment and stabilizes microvascular homeostasis; and the central regulatory control of STAT1, which breaks the vicious cycle of inflammation while uniquely promoting endogenous hyaluronan-mediated tissue repair and lubrication. The high concordance between virtual gene perturbations and Teriflunomide-induced cellular responses, as validated by the AetherCell framework, underscores the reliability of these identified targets. Collectively, this study establishes a scientific basis for the clinical translation of Teriflunomide as a multi-target therapeutic strategy for moderate-to-severe dry eye disease, addressing both pathogenic mechanisms and symptomatic burden. It further highlights the utility of computational pharmacology in dissecting complex disease processes and advancing precision medicine in ocular surface disorders.

## Figures and Tables

**Table 1:** Molecular docking binding affinities of Teriflunomide with key target proteins.

Target Protein	Affinity (kcal/mol)
CTSS	−8.77
STAT1	−7.59
PTGS1	−6.62
TOP2A	−6.00
PSMB9	−5.34
CDK1	−4.97

## Data Availability

The bulk RNA-seq datasets analyzed in this study were obtained from the Gene Expression Omnibus (GEO) database under accession numbers GSE149832, GSE186015, GSE208297, GSE252984, GSE272701, GSE306385, and GSE316045. The single-cell RNA-seq (scRNA-seq) data were retrieved from GEO under the accession number GSE132420. All these datasets are publicly available and can be accessed through the NCBI website.

## Supplementary Materials

The following supporting information can be downloaded at: https://www.mdpi.com/article/10.3390/cimb48050492/s1.
