# Supplementary material for: Molecular Target Discovery and Systemic Mechanism Analysis of Teriflunomide for Dry Eye Disease
Source: Curr Issues Mol Biol. 2026 May 9;48(5):492. doi: 10.3390/cimb48050492 (PMC13206075; doi:10.3390/cimb48050492)
Supplement: Supplementary file 1 [file cimb-48-00492-s001.zip › Supplementary.pdf]

1 Supplementary Figures

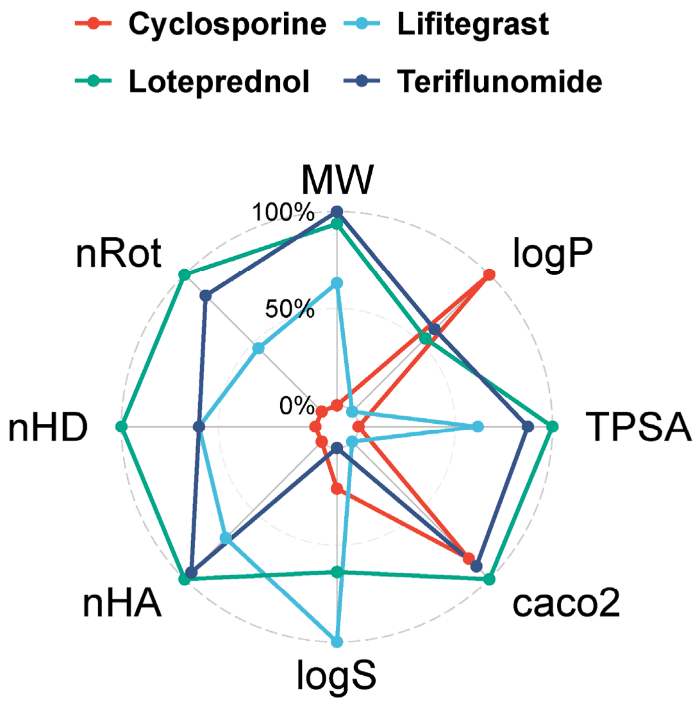

Supplementary Figure S1. Comparison of physicochemical properties and ADMET profiles. The radar chart illustrates the drug-likeness and pharmacokinetic potential of Teriflunomide versus clinical DED drugs across the following parameters: MW (Molecular Weight): Affects molecular mobility and permeability across ocular tissues. LogP / LogS: Indicators of lipophilicity and aqueous solubility, crucial for corneal penetration and formulation stability. TPSA (Topological Polar Surface Area): Correlates with the drug's ability to permeate cell membranes. Caco-2: Predicts the efficiency of intestinal or ocular epithelial membrane transport. nHA / nHD: Number of hydrogen bond acceptors and donors, which influence binding affinity and solubility. nRot: Number of rotatable bonds, representing the molecular flexibility and its impact on binding entropy.

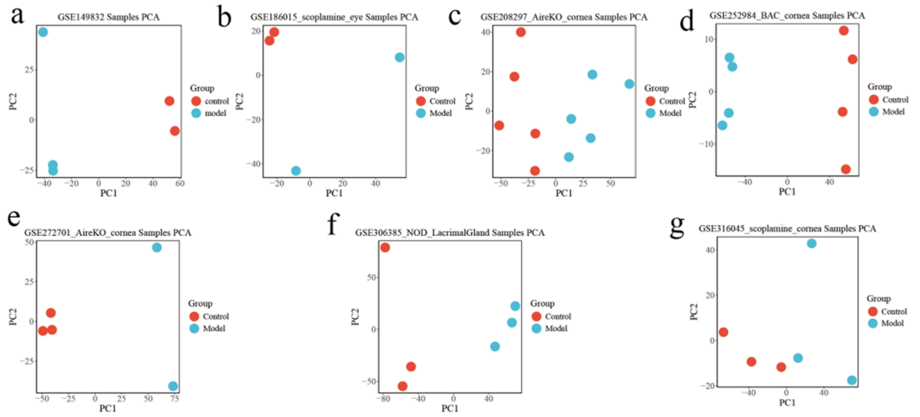

Supplementary Figure S2. Principal Component Analysis (PCA) of seven independent DED-related datasets. Individual PCA plots for each of the datasets used in the integrative analysis are shown: (a) GSE149832, (b) GSE186015, (c) GSE208297 , (d) GSE252981, (e) GSE272700, (f) GSE306585, and (g) GSE316045. In each plot, samples are color-coded by group (Red: Control, Blue: DED Model). Clear separation along the first principal component (PC1) is observed in most datasets, indicating that disease state represents the primary axis of variation in these studies.

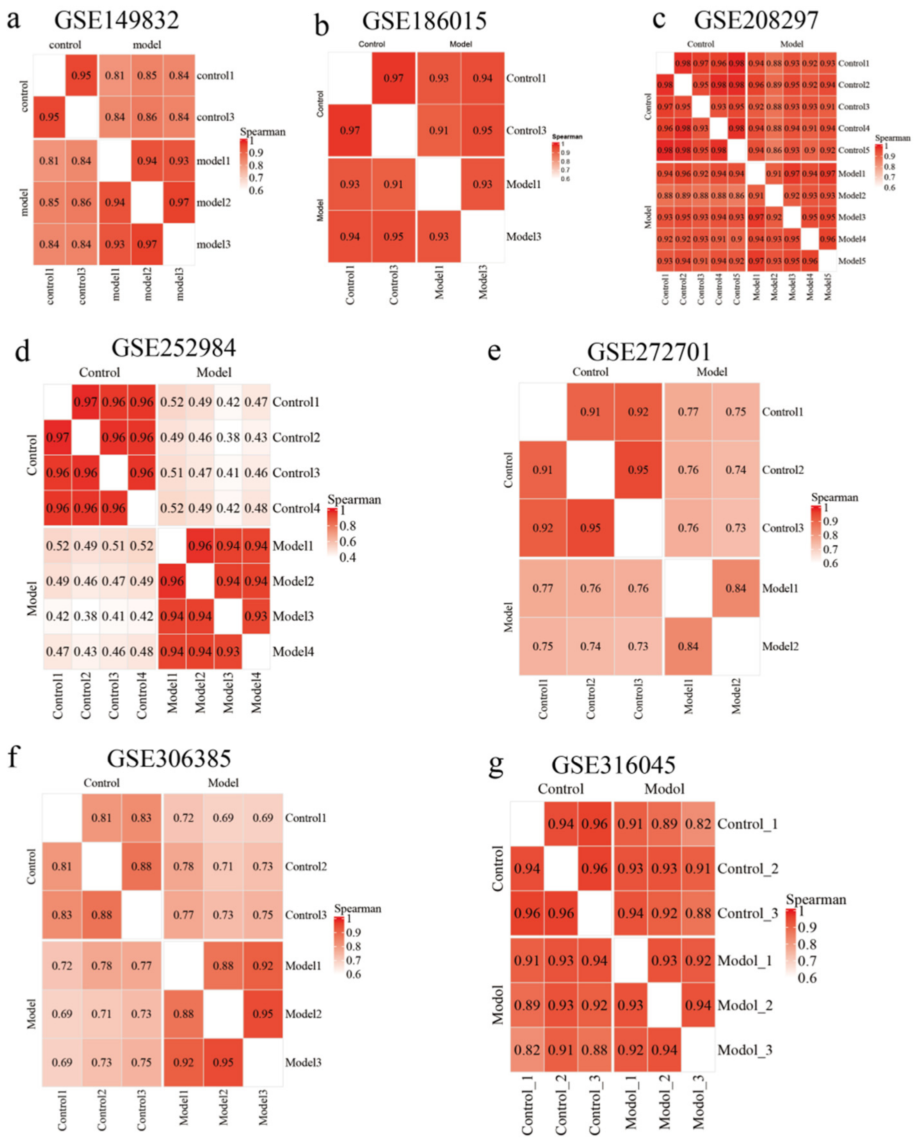

Supplementary Figure S3. Sample correlation analysis of seven independent DED-related datasets. Heatmaps displaying the pairwise Spearman correlation coefficients between samples within each dataset: (a) GSE149832, (b) GSE186015, (c) GSE208297, (d) GSE252984, (e) GSE272701, (f) GSE306385, and (g) GSE316045. The color intensity and numerical values indicate the strength of the correlation (ranging from 0.4 to 1.0). High intra-group correlation and distinct inter-group patterns demonstrate the data quality and biological consistency within each study, supporting their suitability for integrated transcriptomic analysis.

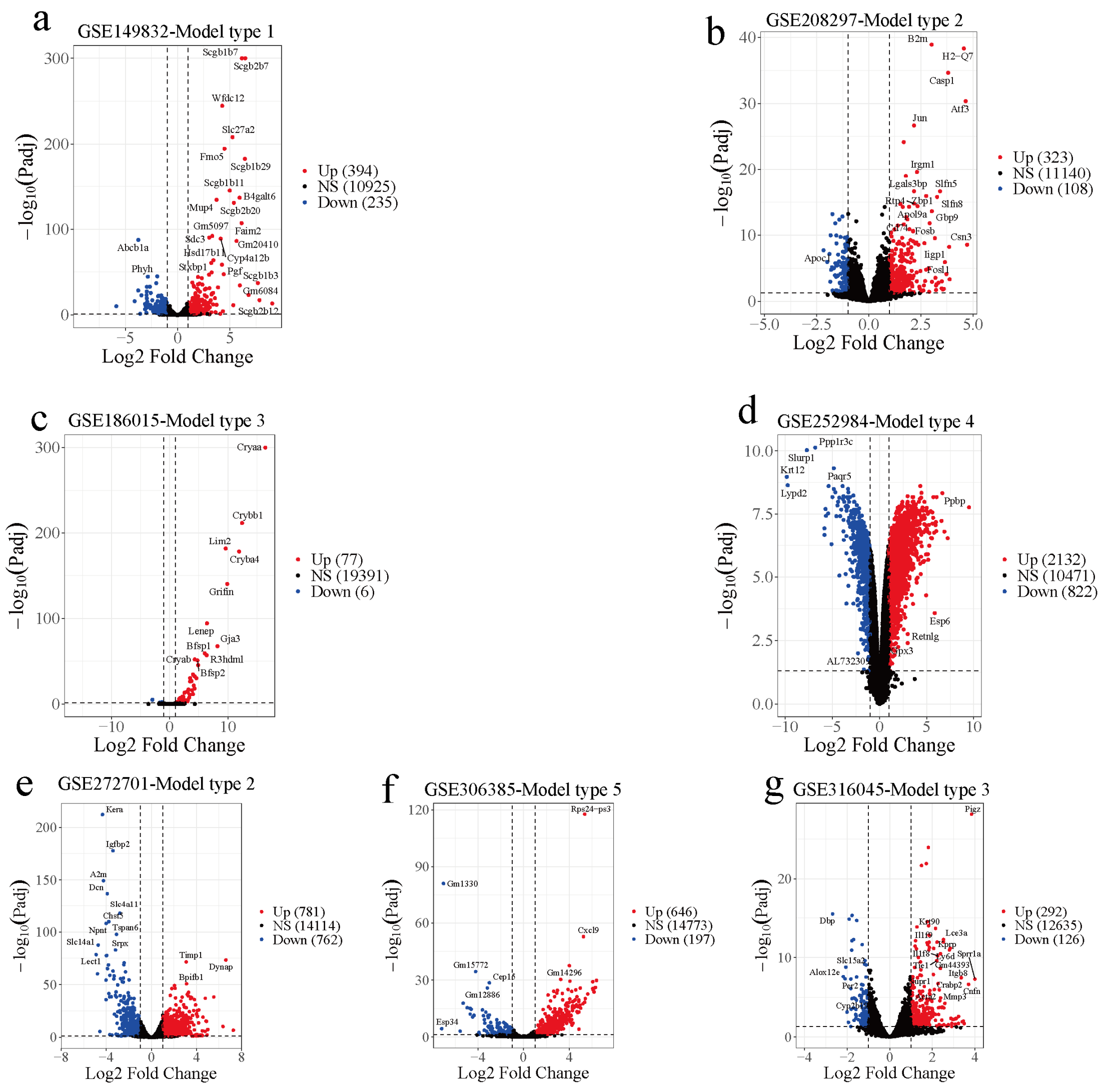

Supplementary Figure S4. Volcano plots of differentially expressed genes across seven independent dry eye disease (DED) transcriptomic datasets. Differential expression analysis was performed for seven mouse DED models representing five distinct induction mechanisms. (a) GSE149832, Model type 1: genetic water transport defect (AQP5-/-). (b) GSE208297, Model type 2: immune tolerance deficiency (Aire-/-). (c) GSE186015, Model type 3: pharmacological secretory block (scopolamine-induced). (d) GSE252984, Model type 4: direct corneal epitheliotoxicity (benzalkonium chloride-induced). (e) GSE272701, Model type 2: immune tolerance deficiency (Aire-/-). (f) GSE306385, Model type 5: Sjögren's-like dacryoadenitis (NOD spontaneous autoimmune exocrinopathy). (g) GSE316045, Model type 3: pharmacological secretory block (scopolamine-induced). Red dots indicate significantly up-regulated genes, blue dots indicate significantly down-regulated genes, and black dots represent non-significant genes. The x-axis represents log2 fold change (log2FC), and the y-axis represents −log10(adjusted P value).

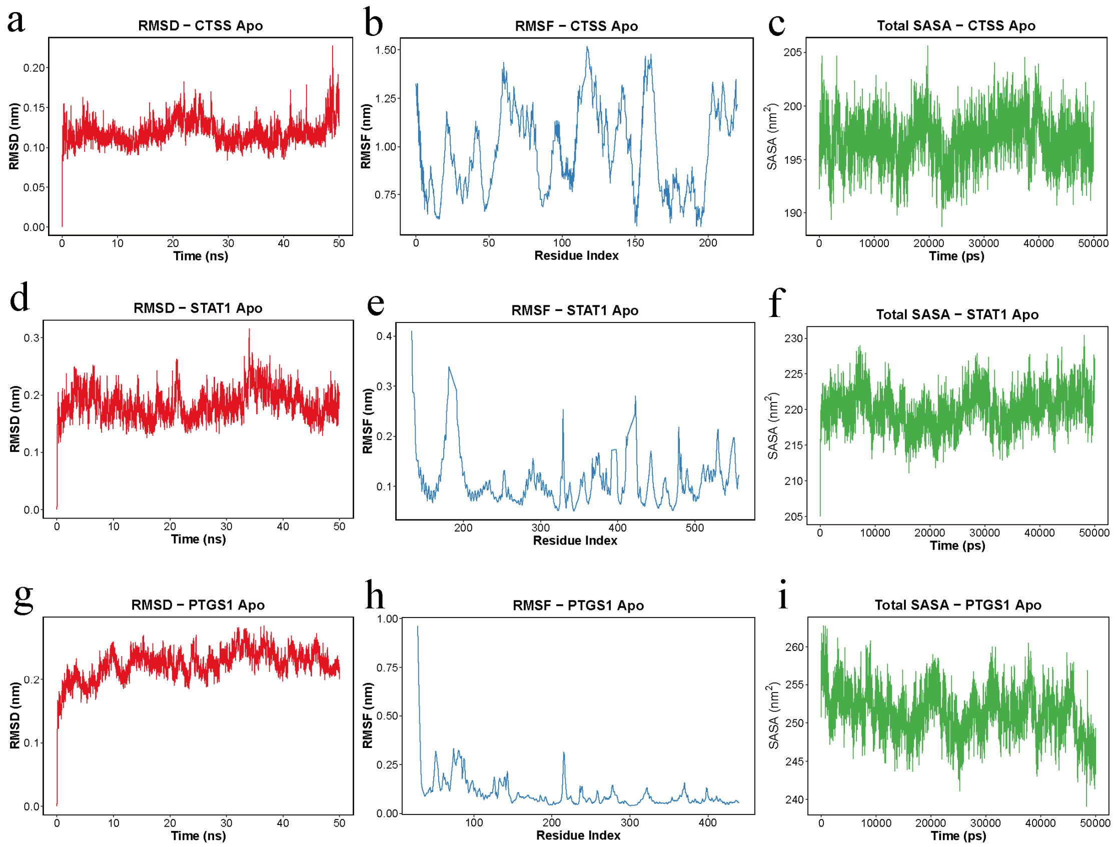

Supplementary Figure S5. Molecular dynamics simulation analysis of apo (ligand-free) proteins CTSS, STAT1, and PTGS1 over 50 ns. (a, d, g) RMSD profiles showing the overall structural stability of each apo protein over time. (b, e, h) RMSF plots illustrating residue-level flexibility of apo proteins. (c, f, i) SASA profiles reflecting solvent-accessible surface area changes in apo systems during the simulation.

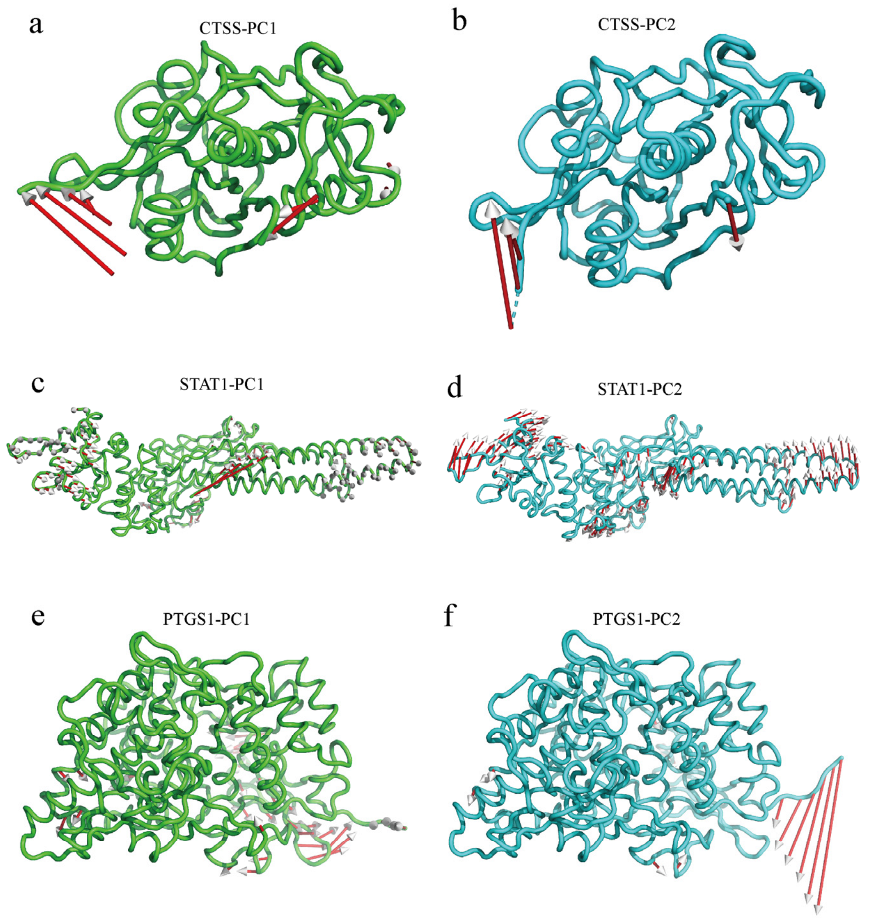

Supplementary Figure S6. Principal component analysis (PCA) of ligand-bound protein complexes (CTSS, STAT1, and PTGS1). (a, c, e) PC1 modes (green) representing the dominant collective motions of each protein. (b, d, f) PC2 modes (cyan) illustrating secondary conformational fluctuations. Red arrows indicate the direction and magnitude of dominant motions derived from the eigenvectors. PC1 primarily captures global or collective motions, while PC2 reflects more localized or alternative dynamic modes.

2 Supplementary Tables

Supplementary Table S1. Identification of 886 core leading-edge genes. > This table summarizes the 886 core leading-edge genes derived from our multi-omics integration and GSEA analysis. These genes represent the key drivers of the prioritized biological pathways involved in dry eye disease pathogenesis.

Supplementary Table S2. Virtual screening of Teriflunomide targets via SuperPred. > To explore the pharmacological mechanism of Teriflunomide, we employed the SuperPred platform for target prediction. This table provides a comprehensive list of 113 predicted

protein targets, which served as the basis for subsequent molecular docking and virtual cell-based validation.
